# Plant cell wall profiling by fast maximum likelihood reconstruction (FMLR) and region-of-interest (ROI) segmentation of solution-state 2D ^1^H–^13^C NMR spectra

**DOI:** 10.1186/1754-6834-6-45

**Published:** 2013-04-26

**Authors:** Roger A Chylla, Rebecca Van Acker, Hoon Kim, Ali Azapira, Purba Mukerjee, John L Markley, Véronique Storme, Wout Boerjan, John Ralph

**Affiliations:** 1 DOE Great Lakes Bioenergy Research Center, The Wisconsin Energy Institute, 1552 University Avenue, Madison, WI, 53726, USA; 2 Department of Biochemistry, University of Wisconsin Madison, 433 Babcock Drive, Madison, WI, 53706, USA; 3 Department of Plant Systems Biology, Flanders Institute for Biotechnology (VIB), Technologiepark 927, Ghent, 9052, Belgium; 4 Department of Plant Biotechnology and Bioinformatics, Ghent University, Technologiepark 927, Ghent, 9052, Belgium

**Keywords:** Lignin composition, Spectral deconvolution, Maximum likelihood, NMR spectroscopy, Multivariate data analysis

## Abstract

**Background:**

Interest in the detailed lignin and polysaccharide composition of plant cell walls has surged within the past decade partly as a result of biotechnology research aimed at converting biomass to biofuels. High-resolution, solution-state 2D ^1^H–^13^C HSQC NMR spectroscopy has proven to be an effective tool for rapid and reproducible fingerprinting of the numerous polysaccharides and lignin components in unfractionated plant cell wall materials, and is therefore a powerful tool for cell wall profiling based on our ability to simultaneously identify and comparatively quantify numerous components within spectra generated in a relatively short time. However, assigning peaks in new spectra, integrating them to provide relative component distributions, and producing color-assigned spectra, are all current bottlenecks to the routine use of such NMR profiling methods.

**Results:**

We have assembled a high-throughput software platform for plant cell wall profiling that uses spectral deconvolution by Fast Maximum Likelihood Reconstruction (FMLR) to construct a mathematical model of the signals present in a set of related NMR spectra. Combined with a simple region of interest (ROI) table that maps spectral regions to NMR chemical shift assignments of chemical entities, the reconstructions can provide rapid and reproducible fingerprinting of numerous polysaccharide and lignin components in unfractionated cell wall material, including derivation of lignin monomer unit (S:G:H) ratios or the so-called SGH profile. Evidence is presented that ROI-based amplitudes derived from FMLR provide a robust feature set for subsequent multivariate analysis. The utility of this approach is demonstrated on a large transgenic study of *Arabidopsis* requiring concerted analysis of 91 ROIs (including both assigned and unassigned regions) in the lignin and polysaccharide regions of almost 100 related 2D ^1^H–^13^C HSQC spectra.

**Conclusions:**

We show that when a suitable number of replicates are obtained per sample group, the correlated patterns of enriched and depleted cell wall components can be reliably and objectively detected even prior to multivariate analysis. The analysis methodology has been implemented in a publicly-available, cross-platform (Windows/Mac/Linux), web-enabled software application that enables researchers to view and publish detailed annotated spectra in addition to summary reports in simple spreadsheet data formats. The analysis methodology is not limited to studies of plant cell walls but is amenable to any NMR study where ROI segmentation techniques generate meaningful results.

Please see Research Article: http://www.biotechnologyforbiofuels.com/content/6/1/46/.

## Background

Interest in the detailed lignin and polysaccharide composition of plant cell walls has surged within the past decade partly as a result of biotechnology research aimed at converting biomass to biofuels [1,2]. Numerous studies have established the link between the relative amount of lignin and cellulose in vascular tissues and the accessibility of plant cell walls to chemical, enzymatic, and microbial digestion [2-4]. Comparisons of different species [5], and transgenic studies in which synthesis of cell wall components is genetically modified [3,4,6], are particularly useful in identifying these linkages.

High-resolution, solution-state 2D ^1^H–^13^C HSQC NMR spectroscopy has proven to be an effective tool for rapid and reproducible fingerprinting of the numerous polysaccharides and lignin components in unfractionated plant cell wall materials [7-11]. Recent advances in “ball-milled” sample preparations dissolved or swelled in organic solvents have enabled unfractionated material to be profiled without the need for component isolation [12,13]. The heterogeneous and highly polymeric nature of the ball-milled cell wall material, in which polymers are of significantly lower degree of polymerization (DP) than in the intact cell wall (where DP of cellulose is ~7000-15000) [13], results in spectra with broad linewidths and considerable complexity. However, the dispersion provided by the two-dimensional correlation of protons to their attached ^13^C nuclei, at natural abundance, enables resolution and assignment of numerous lignin, cellulose, and hemicellulosic components. The 2D ^1^H–^13^C HSQC experiment is thus a powerful tool for cell wall profiling based on our ability to simultaneously identify and comparatively quantify numerous components within spectra generated with relatively short acquisition times (15–20 min/sample, but up to 5 h if excellent signal-to-noise and the ability to detect minor components is desirable).

As sample preparation and data acquisition methods have improved [10,11], the task of spectral analysis has become a bottleneck in large studies. NMR-based chemometrics is one data analysis approach recently applied to investigate structural/compositional differences between wood samples from *Populus*[14]. Chemometrics is a multivariate approach with an extensive history in metabonomics [15,16]. General strengths of a multivariate approach that simultaneously examines features from different sample groups include the ability to detect subtle patterns among features across sample groups, albeit sometimes with confusion by artifacts [12], and assess the relative importance of each feature for group discrimination [14].

NMR-based chemometrics is characterized by a sequence of steps involving: i) NMR data processing, including baseline correction if necessary; ii) generation of a feature set usually by selecting intensity values on each peak or summing over segmented regions (spectral binning); iii) production of a data table in which each sample represents a row and the features are columns; iv) normalization (row-based) and scaling (column-based) of the data; and v) multivariate statistical modeling. The greatest pitfalls lie in feature selection (step ii). Originally developed as a rapid and consistent method to generate data sets automatically and handle problems of peak “drift”, spectral binning unfortunately reduces spectral resolution and can generate artifacts in crowded spectra where the boundary of a bin may lie at the center of a signal. Even when the full resolution spectrum is used without binning, the common technique of analyzing 2D data by generating a 1D row vector from the 2D grid results in a loss of correlation information between the ^1^H and ^13^C intensity values during the analysis process, although this may be retained by indexing the 1D data so that 2D spectra can be recreated, including after, for example, principal component analysis [14].

An alternative to peak-based or bin-based feature selection is to mathematically model the data and use the modeled parameters as features for subsequent analysis. If the model can efficiently represent the relevant features of the data, the modeling step dramatically reduces the number of columns in the data matrix (data reduction) without loss of relevant information or generation of artifacts. Recently, spectral deconvolution using fast maximum-likelihood reconstruction (FMLR) was shown to accurately quantify metabolites in 2D ^1^H–^13^C HSQC spectra [17,18]. FMLR constructs the simplest time-domain model (e.g., the model with the fewest number of signals and parameters) whose frequency spectrum matches the visible regions of the spectrum obtained from identical Fourier processing of the data [19,20].

Spectral analysis of 2D ^1^H–^13^C HSQC NMR data by FMLR would appear to be an attractive approach for high-throughput plant cell wall profiling in the following respects:

i. FMLR has already been shown to accurately model the characteristics of complex 2D ^1^H–^13^C HSQC solution spectra [17], and can be performed with minimal input information and operator intervention (moderately high throughput).

ii. Because of the high spectral dispersion inherent in 2D ^1^H–^13^C NMR data, the detailed but localized amplitude and frequency information derived from FMLR should be easily combinable with assigned region-of-interest tables to generate the relative concentration of cell wall components in each sample (cell wall component profiles). Previous work has shown the utility of region of interest (ROI)-segmentation in quantitative 2D ^1^H–^13^C NMR studies [21,22].

iii. ROIs that correspond to a resolved peak or peak cluster can be defined even when the NMR assignment is tentative or unknown. The cell wall component profiles are thus suitable for both untargeted and targeted profiling.

iv. Simple visual inspection of the cell wall component profiles might suffice to identify patterns of enrichment and depletion of various components between sample groups.

v. The cell wall component profiles are also a robust feature set for input into multivariate analysis.

We apply here the spectral analysis methodology of FMLR with ROI-based segmentation to a large (98 samples) 2D ^1^H–^13^C NMR study of *Arabidopsis* lignin mutants and controls involving 20 sample groups (10 consolidated groups)*.* Our focus here is not on biological conclusions to be drawn from the study (this is published concomitantly) [23], but on the methodology and software implementation of data analysis for powerful cell wall profiling by NMR.

## Materials & methods

### Biological sources

For ten genes involved in lignin biosynthesis [24], two *Arabidopsis thaliana* mutant alleles were analyzed (see Table 1). The 20 sample groups were consolidated into 10 effective sample groups based on statistically similar lignin composition. These samples were drawn from an overall pool of forty biological replicates of each homozygous mutant and 32 biological replicates for wild-type type were grown simultaneously in a random block design, spread over different trays, in the same environment. Plants were grown first under short-day conditions (8 h light, 21°C, humidity 55%) during 6 weeks, and then transferred to the greenhouse. For all of the biological repeats, the main stem was harvested just above the rosette when the plant was completely senesced. Once harvested, axillary inflorescences, siliques and seeds, as well as the bottom 1 cm of the main stem, were removed. The rest of the inflorescence stem was cut into 2 mm pieces and biological repeats were pooled per 8 stems to obtain 5 biological replicates for the mutant alleles and 4 repeats for the wild-type, except for *c4h-2*, *ccr1-3*, and *ccr1-6*. In order to have enough biomass for NMR analyses, the senesced inflorescence stems of *c4h-2* were pooled in one single pool, for *ccr1-3* the stems were pooled in 3 pools, and for *ccr1-6* in 4 pools.

**Table 1:** **Sample groups of *Arabidopsis thaliana *used in study**

**Gene**	**Sample groups(s)**	**# replicates**	**WT-eff**
WT	WT	4	X
*pal1*	*pal1-2, pal1-3*	10 (5,5)	X
*pal2*	*pal2-2, pal2-3*	10 (5,5)	X
*c4h*	*c4h-2 , c4h-3*	6 (1,5)	
*4cl1*	*4cl1-1, 4cl1-2*	10 (5,5)	
*4cl2*	*4cl2-1, 4cl2-2*	10 (5,5)	X
*ccoaomt1*	*ccoaomt1-3, ccoaomt1-5*	11 (6,5)	
*ccr1*	*ccr1-3, ccr1-6*	7 (3,4)	
*f5h1*	*f5h1-2, f5h1-4*	10 (5,5)	
*comt*	*comt-1, comt-4*	10 (5,5)	
*cad6*	*cad6-1, cad6-4*	10 (5,5)	X

The table lists the ten genes [24] involved in the study and the associated sample groups. The two mutant alleles associated with the same gene were consolidated into a single sample group for the purposes of this study. This consolidation was justified in terms of a calculated *t*-test value between the two groups that was less than 3 for each normalized %S, %G, and %H value. The designation of “WT-eff” refers to a sample group whose percentage S, G, and H values are not statistically different from those of the wild-type group.

### Sample preparation and cell wall dissolution

Preparation of whole cell wall samples for NMR was largely as described previously [8,10]. In brief, pre-ground *Arabidopsis* stem samples (~200 mg) were extracted with water (3×) and then 80% aqueous ethanol (sonication 3 × 20 min) yielding 70–100 mg of cell wall material. Isolated cell walls (~80 mg) were ball-milled (4 × 30 min milling and 5 min cooling cycles, total time 2 h 20 min) using a Fritsch (Idar-Oberstein, Germany) Planetary Micro Pulverisette 7 ball mill vibrating at 800 rpm with 12 mL ZrO_2_ vessels containing thirty 5 mm ZrO_2_ ball bearings. Aliquots of the ball-milled whole cell walls (~60 mg) were transferred into NMR sample tubes, swollen in DMSO-d_6_:pyridine-d_5_ (4:1, v/v, 600 μl), and subjected to 2D NMR experiments.

### Analysis overview

The process of FMLR reconstruction with ROI segmentation can be viewed as a sequence of steps involving:

1. NMR data acquisition and processing

2. Ensemble matrix formation and importation of grouping information

3. Spectral normalization

4. ROI segmentation

5. Spectral deconvolution by FMLR

6. ROI assignment and generation of a feature matrix

7. ROI normalization of the feature matrix

8. Statistical analysis of the features

### NMR data acquisition and processing

NMR spectra were acquired on a Bruker Biospin (Billerica, MA) AVANCE 700 MHz spectrometer fitted with a cryogenically cooled 5-mm TXI gradient probe with inverse geometry (proton coils closest to the sample). Cell wall samples were swollen in 4:1 DMSO-d_6_:pyridine-d_5_, 0.5 mL; the central DMSO solvent peak was used as internal reference (δ_C_, 49.5; δ_H_, 3.49 ppm). Adiabatic HSQC experiments (hsqcetgpsisp.2.2) were carried out using the parameters described previously [10].

The initial steps of NMR data processing (conversion from time-domain to frequency domain) were performed using Topspin 3.1-Macintosh (Bruker Biospin, Rheinsteten, Germany). The processing consisted of i) apodization (matched Gaussian in F2, squared cosine-bell in F1), ii) zero-filling, iii) Fourier transformation, and iv) phase correction; no linear prediction was used.

The apodization and zero-filling parameters associated with steps i-iv along each dimension *d* define a vector operator ${\hat{F}}_{d}$ that can be applied identically to both the acquired FID and the model FID along dimension *d*. In the FMLR algorithm, the ${\hat{F}}_{d}$ operator converts discrete basis functions in the time domain (see Table 2) to discrete basis functions in the frequency domain.

**Table 2:** Basis functions and parameters used in FMLR

**Basis functions**					
**Name**	**Type**	**Expression**	**Derivative**	**Usage**	
Sinusoid	Complex	*e*^*iωt*^	*ite*^*iωt*^	Always	
Damping function	Real	$e^{-at^{\eta }}$	$-t^{\eta }e^{-at^{\eta }}$	Used except along indirect dimensions of constant time experiments	
**Parameters**					
**Parameter**	**Symbol**	**Variable**	**Basis function**	**Initial value**	**Constrained**
Frequency	Ω	Yes	Sinusoid	From peak position	No
Decay Rate	Α	Yes	Damping function	From “prototype” signal	Yes
Decay Power	Η	No	Damping function	Assigned based on profiling of data sets. Fixed per analysis on single data set	No (fixed)

The time domain basis functions along each model dimension are the complex product of a sinusoid basis function with a damping function. The corresponding frequency domain functions are obtained from Fourier transformation using a digital operator derived from the acquisition and processing parameters. Multidimensional basis functions are derived from the product of the orthogonal component basis functions along each dimension. For gradient-based (non-linear) optimization of the parameters, the derivative basis functions are used. The exponent η appearing in the decay rate term is a value that modulates the signal between a Lorentzian (η = 1) and Gaussian (η = 2) decay profile. This value is adjusted to fit a similar class of peak shapes and is left constant throughout the optimization of any given data set. The corresponding derivative basis functions are used to calculate the derivative of the basis function with respect to the angular frequency (sinusoid) and decay rate (damping function).

### Ensemble matrix formation

To facilitate concerted analysis of multiple data sets, the 2D absorption spectra (portions remaining after phase correction and discarding of imaginary components) were appended together to form an “ensemble” data set (pseudo-3D matrix). Two of the dimensions correspond to the ^1^H and ^13^C spectral frequencies and the remaining dimension is a “pseudo-dimension” that encodes the spectral index (and identity of the sample source).

### Spectral normalization

The intensity of each data point in the spectrum was normalized to the sum of all intensity points prior to spectral analysis. This pre-analysis normalization step removes intensity modulation due to varying concentrations of biological material and allows the same intensity thresholds to be applied across all data sets.

### ROI segmentation

**Figure 1:**
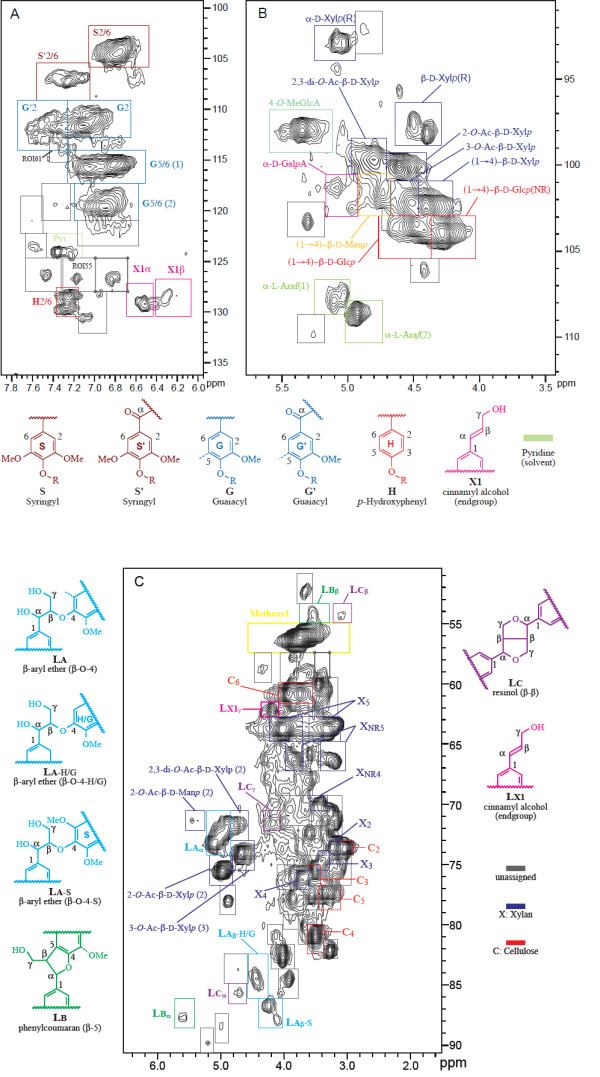
**Annotated high-resolution, solution-state 2D **^**1**^**H–**^**13**^**C HSQC NMR of a wild-type *Arabidopsis *spectrum in the A) lignin aromatic, B) polysaccharide anomeric, and C) lignin-polysaccharide regions.** The rectangular boxes denote ROIs that correspond to assigned NMR transitions (colored boxes with annotations) or simply resolved regions of the spectrum that have yet to be assigned (gray boxes). The unassigned regions are associated with an ID that is used to identify them in the feature matrix. To avoid crowding the figure, the ID does not appear as a label. The lowest contour in the figure corresponds to an intensity level of 3 SD of rms noise.

A region of interest (ROI) as used in this context refers simply to a 2D spectral window or “box” associated with a spectral transition from a molecular entity. Regions of interest were manually defined for 91 ROIs within Newton by drawing boxes overlaid on the spectra (see graphical view in Figure 1A-C). Results from previous cell wall profiling studies [8,10,11,25] and model compounds were used to determine the footprint of the ROIs appearing in the figures and to assign 52/91 ROIs in the various spectral regions. As an ROI is drawn once and can be superimposed onto any spectrum, the time required to define their boundaries is based only on the number of ROIs, rather than the number of spectra.

For future studies, ROIs defined from earlier studies can be imported and graphically adjusted to align with the local spectra.

### Fast maximum-likelihood reconstruction (FMLR)

The detailed theory and equations for applying the maximum-likelihood method to analysis of NMR data have been reported previously [19,20], and most recently for the analysis of 2D ^1^H–^13^C data sets in a metabolomics context [17]. The specific steps for performing spectral deconvolution of the *Arabidopsis* data in this study consisted of:

1. Prototype Signal Generation: An isolated signal was graphically selected by the operator as an archetypal signal. The signal giving rise to the peak was fitted using a model whose basis functions and model parameters are specified in Table 2. The decay rate (linewidths) obtained from this optimization were used as initial values for further modeling. For the *Arabidopsis* study, the prototype linewidth was 80 Hz along both the ^1^H and ^13^C dimensions.

2. Constraint Specification: The FMLR algorithm uses constraints on linewidth to assist in convergence of the fitting algorithm in crowded spectral areas. Linewidth constraints are specified as a multiple of the prototype linewidth along each dimension. For the study reported here, the linewidth was constrained to be a factor of 1/2 to 2 relative to the prototype linewidth, i.e., 40–160 Hz.

3. Choosing Noise Thresholds: During spectral deconvolution (see below), signals are added incrementally in a series of iterations. Initially the pick threshold is set to the maximum peak height and is then reduced geometrically by a factor of $\sqrt{2}$ at the conclusion of each iteration. The analysis algorithm is terminated when the pick threshold reaches a minimum value specified as a multiple of signal-to-noise. The S/N threshold for this study was 4.0.

4. Spectral Deconvolution: To avoid modeling extraneous features of the spectrum, only those peaks in a spectrum contained within at least one ROI were modeled by spectral deconvolution. Spectral deconvolution was initiated after steps 1–3 above and continued without operator intervention for a series of 10 iterations that yielded 22,389 signals (5 × 22,389 = 111,945 total parameters) across the 98 data sets. The total duration time of the analysis was 28 minutes on an off-the-shelf Pentium laptop [AMD Phenom II N870 Triple-Core Processor 2.3 GHz, 6.0 GB RAM, Windows 7 SP 1 2009 64 bit OS, Java 1.6.0_25_b06 with Java Hot Spot (TM) 64 bit server virtual machine].

### ROI assignment and feature matrix generation

A signal was assigned to a target ROI if its peak center existed within the boundaries of that ROI. When a source peak is contained within more than one target ROI (i.e., two or more target ROIs overlap), the Newton assignment algorithm assigns the source peak to the target ROI with the greatest “gravity metric” (product of source peak and target peak intensities divided by the spectral distance between the source and target peak summed over all target peaks).

The amplitude of each ROI was calculated as the simple sum of all signal amplitudes (obtained from spectral deconvolution) assigned to that ROI. From this information, a “feature matrix” can be constructed of a 2D *n*_*r*_ × *n*_*s*_ matrix where *n*_*r*_ is the number of regions of interest and *n*_*s*_ is the number of spectra.

### ROI normalization

After generation of the feature matrix, which can be imported into any standard spreadsheet program (csv file format), the value of each ROI amplitude (i.e., the sum of amplitudes of all signals located within the region of interest) was normalized by a value *L* representing lignin content in the spectrum. The value *L* is the weighted sum of integrals of the following ROI amplitudes:

(1) $$L=\left[S2/6\right]+\left[S'2/6\right]+2\left[G2\right]+2\left[G2'\right]+\left[H2/6\right]$$

Where [*S*2/6], [*S*'2/6], [*G*2], [*G*'2], [*H*2/6] represent the ROI amplitudes in regions corresponding to the S (syringyl), G (guaiacyl), and H (*p*-hydroxyphenyl) lignin types [See also Figure 1A]. The coefficients are derived from the relative ratio of proton/carbon pairs assigned to the spectral regions. This normalization step produces a meaningful metric (i.e., as a fraction of lignin content in the sample) for reporting the amplitudes of cell wall components. The normalization operation was performed within a spreadsheet program (Microsoft Excel).

For spectra in which an internal standard (e.g., DSS or formate) is present at a fixed concentration (not shown here), the software also supports normalization by the intensity of the ROI associated with the internal standard.

### Statistical data analysis

Differences in ROI amplitudes between *Arabidopsis* mutant lines and a wild type were analyzed with analysis of variance using the glm procedure of the SAS/STAT software, Version 9.3 of the SAS System for windows. Copyright © 2011, SAS Institute Inc., Cary, NC, USA. P-values were adjusted for multiple testing using the Dunnett approach. All reported significant differences are at the overall α level of 0.05.

### Data visualization

All of the contour plots contained in the figures here were rendered by Newton and exported in the vector-based format of encapsulated postscript (EPS). Annotations were added using Adobe Illustrator. Bar charts and similar graphics comparing ROI amplitudes were produced by Microsoft Excel and SAS.

### Software availability

The software application can be downloaded and run from instructions found at http://newton.nmrfam.wisc.edu/. The host machine must have an installed version of the Java Runtime Environment (JRE) v1.6+ to run the application; Microsoft Windows, Apple MacOS, and various Linux implementations are all supported.

## Results and discussion

### Region of interest specification

After processing the spectra and creating the ensemble, a set of 91 ROIs were specified as 2D rectangles along the ^1^H and ^13^C axis as shown in the lignin, lignin-polysaccharide, and polysaccharide-anomeric regions of Figure 1A-C. The spectral regions shown in each figure were obtained from a selected spectrum from the wild-type sample group of *Arabidopsis*. The boundaries were graphically drawn to segment the spectrum into clusters of signals that are resolved from one another (although the signals *within* a cluster may be only partially resolved). Assignments of plant cell wall components from previous studies [10,11] using model compounds were used to assign 52 of the 91 ROIs (see Figure 1A-C). Once specified for a given study, a ROI table can be exported and imported into other studies with minimal adjustment.

### Spectral deconvolution by FMLR

A mathematical model of all signals present in the spectral ensemble was obtained by spectral deconvolution using fast maximum likelihood reconstruction (see FMLR section of methods for details). Signals present in an ROI were modeled if the height of the residual peak was at least 4.0 standard deviations (SD) above the measured root-mean-square (rms) noise of the ensemble. Peaks outside of any ROI were ignored. Each signal was modeled with five parameters: a scalar amplitude, a frequency along each dimension, and a decay rate (linewidth) along each dimension. The final statistics associated with the deconvolution are summarized in Table 3.

**Table 3:** Normalized S/G/H lignin changes

**A: Estimated differences from FMLR**									
**Sample group**	**S**			**G**			**H**		
	**Δ%**	**CI**	**p-value**	**Δ%**	**CI**	**p-value**	**Δ%**	**CI**	**p-value**
*pal1*	1.6	-2.1;5.3	0.73	-1.6	-4.8;1.6	0.57	0.03	-3.5;3.6	1
*pal2*	0.7	-3.0;4.4	1	-1.4	-4.6;1.8	0.73	0.6	-2.9;4.2	1
***c4h***	**9.2**	**5.1;13.2**	**3E-7**	**-15.9**	**-19.3;-12.4**	**1E-12**	**6.7**	**2.8;10.6**	**0.0001**
***4cl1***	**10.2**	**6.5;13.9**	**1E-9**	**-13.2**	**-16.3;-10.0**	**1E-12**	3.0	-0.6;6.5	0.13
*4cl2*	-1.1	-4.8;2.6	0.94	0.6	-2.6;3.8	1	0.5	-3.1;4.0	1
***ccoaomt1***	**8.2**	**4.5;11.8**	**5E-7**	**-11.6**	**-14.7;-8.4**	**1E-12**	3.4	-0.1;6.9	0.06
***ccr1***	**-12.5**	**-16.4;-8.6**	**6E-12**	-2.6	-6.0;0.8	0.18	**15.1**	**11.3;18.9**	**1E-12**
***f5h1***	**-25.0**	**-28.7;-21.3**	**1E-12**	**24.9**	**21.7;28.1**	**1E-12**	0.1	-3.4;3.7	1
***comt***	**-21.9**	**-25.6;-18.2**	**1E-12**	**21.3**	**18.2;24.5**	**1E-12**	0.6	-3.0;4.0	1
*cad6*	0.5	-3.2;4.2	1	-0.2	-3.4;2.9	1	-0.2	-3.8;3.3	1
**B: Estimated differences from ROI Integration**									
**Sample group**	**S**			**G**			**H**		
	**Δ%**	**CI**	**p-value**	**Δ%**	**CI**	**p-value**	**Δ%**	**CI**	**p-value**
*pal1*	1.1	-2.2;4.5	0.90	-1.3	-3.9;1.4	0.65	0.1	-2.4;2.7	1
*pal2*	0.2	-3.1;3.5	1	-0.7	-3.4;2.0	0.98	0.5	-2.1;3.0	1
***c4h***	**7.7**	**4.0;11.3**	**2E-6**	**-12.6**	**-15.5;-9.6**	**1E-12**	**4.9**	**2.2;7.7**	**7E-5**
***4cl1***	**8.2**	**4.9;11.5**	**4E-8**	**-10.3**	**-13.0;-7.6**	**1E-12**	2.1	-0.4;4.6	0.14
*4cl2*	-1.0	-4.4;2.3	0.93	0.6	-2.1;3.3	0.99	0.4	-2.1;3.0	1
***ccoaomt1***	**6.5**	**3.2;9.8**	**1E-5**	**-8.9**	**-11.6;-6.3**	**1E-12**	2.5	-0.05;5.0	0.06
***ccr1***	**-9.6**	**-13.1;-6.1**	**2E-9**	-0.5	-3.4;2.3	1	**10.1**	**7.4;12.8**	**1E-12**
***f5h1***	**-21.3**	**-24.7;-18.0**	**1E-12**	**21.3**	**18.6;23.9**	**1E-12**	0.07	-2.5;2.6	1
***comt***	**-16.7**	**-20.0;-13.4**	**1E-12**	**16.4**	**13.7;19.1**	**1E-12**	0.3	-2.2;2.9	1
*cad6*	-0.2	-3.5;3.1	1	0.3	-2.4;3.0	1	-0.1	-2.6;2.4	1

‘Δ%’ The average predicted differences in the mean percent of S, G, and H lignin between each sample group and the effective wild-type sample group, ‘CI’ the corresponding 95% confidence intervals, ‘p-value’ the Dunnett adjusted p-values. The average predicted differences in Table 3A and Table 3B reflect values calculated from two different ROI quantitative methods: FMLR reconstruction and ROI integration. Values in bold indicate significant differences at an overall significance level of 0.05.

**Figure 2:**
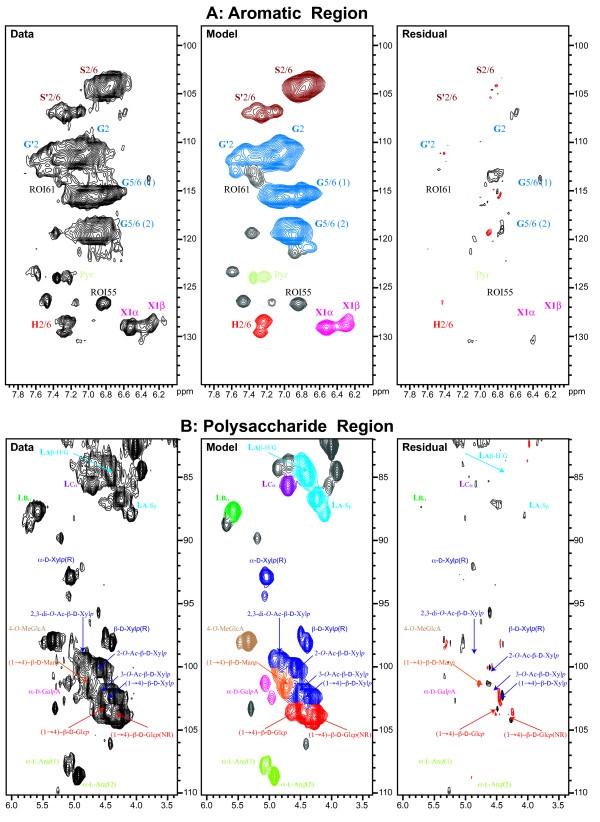
**Processed spectrum (data), FMLR reconstruction (model), and residual of the aromatic (A) and polysaccharide (B) region of the 2D **^**1**^**H–**^**13**^**C HSQC for a wild-type sample of *Arabidopsis*.** The color of a contour is assigned to the color of the ROI associated with the dominant signal in that region. As can be seen in the figure, a minimal number of reconstructed signals is required to yield a model with an associated residual that is less than the noise floor (noise floor = 3.0 SD). The set of contours near (3.6, 76) ppm and (4.7, 63) ppm in (**B**) are not reflective of poor modeling but are a consequence of the fact that no ROI was defined near those positions. Signals in that region of the spectrum were simply not modeled.

The data, model, and residual of spectra from the complex lignin-side-chain plus polysaccharide region of a wild-type sample are shown in Figure 2. Each marker in the figure denotes the center of a signal obtained from spectral deconvolution. Evidence for the suitability of the model to account for major features of the data is that a minimal number of observed signals yields a reconstructed model with a small associated residual (difference between the data and the model). As evident from the figure plotted at a threshold intensity of 3.0 SD, there are few signals in the residual with a peak threshold greater than 3.0 SD (SD of rms noise).

### Feature set of ROI amplitudes

The generation of a meaningful “feature set” of ROI-based amplitudes from FMLR is straightforward. Each peak was automatically assigned to an ROI based on whether its peak center was located within a given ROI (see ROI Assignment section of methods). The amplitude of an ROI was calculated as the simple sum of all signal amplitudes assigned to that ROI. To provide a more meaningful comparison of ROI amplitudes between sample groups, each ROI amplitude was normalized by total lignin content (see ROI Normalization section of Methods). This normalized ROI amplitudes per spectrum results in a feature matrix of 91 ROI amplitudes × 98 spectra (available from Additional Information).

### SGH lignin composition

**Figure 3:**
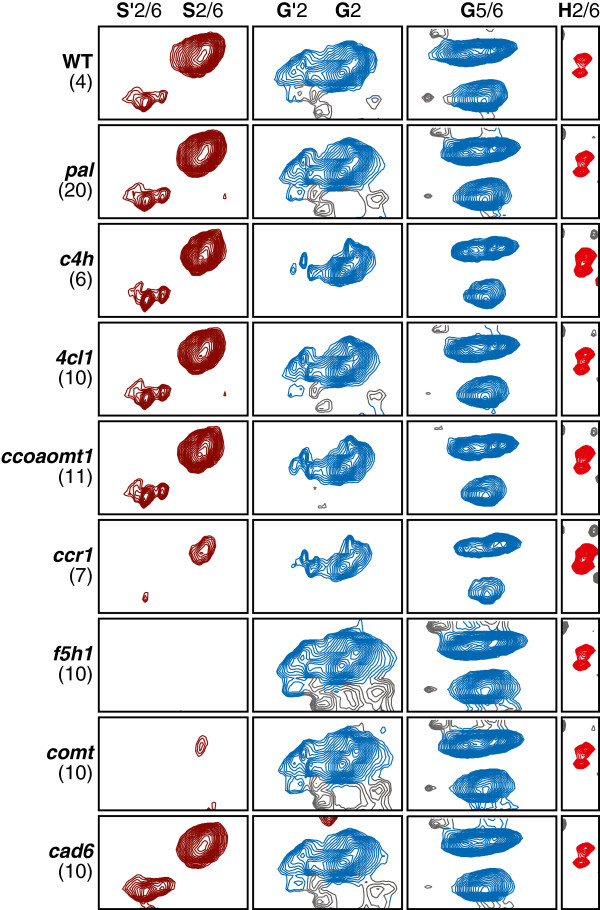
**Contour plots of 2D **^**1**^**H–**^**13**^**C HSQC spectral regions associated with signals assigned to the S′2/6, S2/6, G′2, G2, G5/6, and H2/6 transitions.** The data shown represent the mean spectra of all samples belonging to each sample group (number of spectra for each sample group shown in parentheses). The color of each contour is assigned based on the FMLR reconstructions, i.e., the dominant signal associated with each grid point is used to assign a color to that pixel (and related contour). The contour plots show the ability of the reconstructions to discriminate between assigned (colored) and unassigned (black) signals that partially overlap.

**Figure 4:**
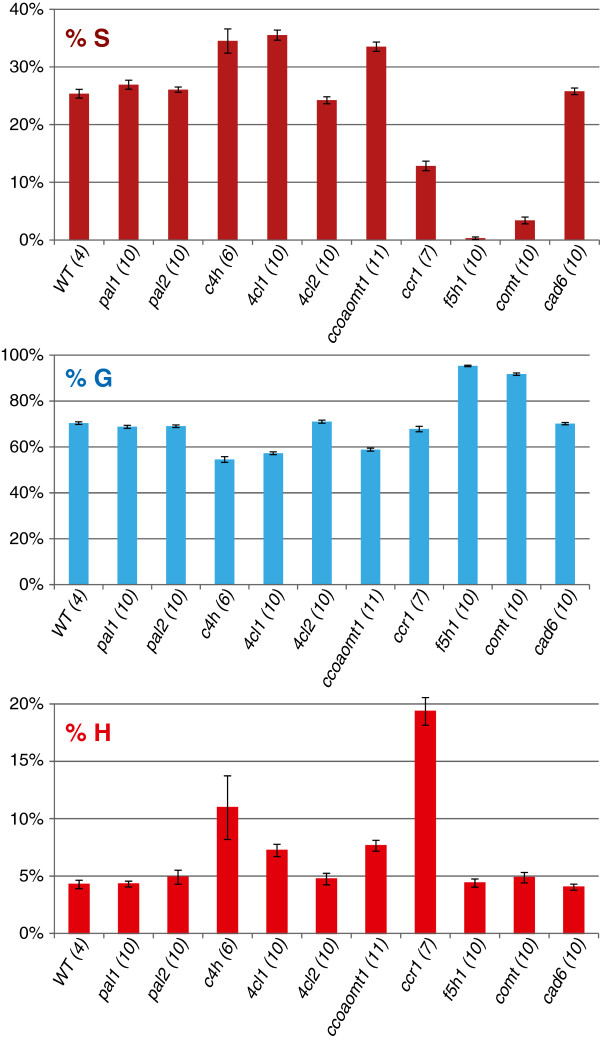
**Bar charts of the mean normalized percentages of S (syringyl), G (guaiacyl), and H (*p*-hydroxyphenyl) lignin units with their standard errors and number of observations (in parentheses).** The values are derived from the ROI feature matrix in which each ROI amplitude is the sum of the amplitude of all modeled signals assigned to that ROI (derived from FMLR, see text for details).

**Figure 5:**
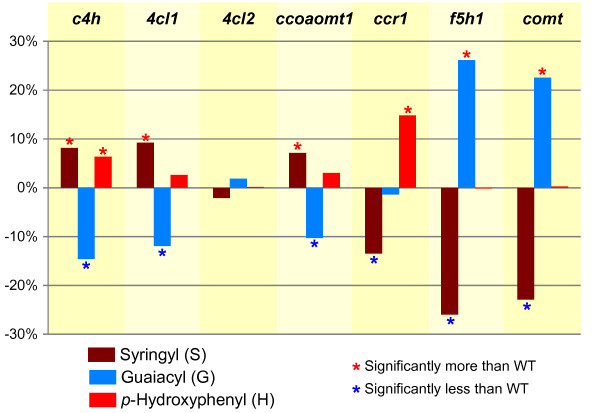
**Bar chart showing pattern of enrichment and depletion of S (syringyl), G (guaiacyl), and H (*p*-hydroxyphenyl) lignin levels (normalized percentages) per sample group.** The *pal* and *cad6* mutants (not shown) showed no significant difference to wild-type. The displayed levels represent the mean predicted difference between each sample group and the effective wild-type sample group.

The relative composition of S (syringyl), G (guaiacyl), and H (*p*-hydroxyphenyl) lignin units is an important element of plant cell wall profiling. The spectral data associated with the SGH ROIs for the sample groups in the study (averaged over all spectra per mutant sample group) is shown as a series of contour plots in Figure 3. In discerning whether relative percentages of SGH lignin are modulated across the sample groups, the bar chart of Figure 4 provides a graphical view of the normalized profiles obtained from the SGH portion of the ROI feature matrix. Differences in S, G, and H percentages between the *Arabidopsis* mutant lines and the wild-type together with Dunnett adjusted p-values are given in Table 3. The overall pattern of enrichment and depletion in the mutant sample groups compared to the wild-types is displayed in the bar chart of Figure 5 where 3 patterns are evident: i) increase of H and S relative to G (*c4h*, *4cl1*, *ccoaomt1*); ii) increase of H relative to S (*ccr1*), and iii) depletion of S relative to G (*f5h1* and *comt*). These results are confirmed by thioacidolysis on the same set of *Arabidopsis* lignin mutants and are published concomitantly [23].

When comparing %S, %G, and %H changes between the mutant groups and wild-type groups, the corresponding p-values are all < 0.0001 (Table 3) for any change greater than 4% (Table 3). The differences are in general larger in magnitude for patterns detected with FMLR reconstruction (Table 3A) versus ROI integration (Table 3B).

### Correlation of ROI changes to SGH modulation

**Figure 6:**
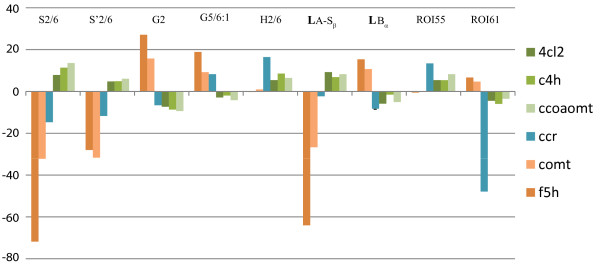
**Bar charts reflecting the correlations between the ROIs and the *Arabidopsis *mutant lines.**

To assess which ROIs might be correlated with the SGH patterns, Pearson correlations were calculated between all ROI amplitudes and the lignin compounds G2, G′2, S2/6, S′2/6, and H2/6. **LA**-Sβ was highly positively correlated to S2/6 (r = 0.94, p < 0.0001) and S′2/6 (r = 0.94, p < 0.0001) and highly negatively correlated to G2 (r = -0.88, p < 0.0001). **LA**-Sβ is assigned specifically to β-syringyl ethers and therefore relates to the S-G distribution, being obviously lower when the S-content is lower. **LB**α is highly positively correlated to G2 (r = 0.82, p < 0.0001). The **LB**α region is assigned to phenylcoumaran (β–5) units in lignins. Such units arise from coupling of a monolignol (at its β-position) with a guaiacyl G (or H) unit (at its 5-position), but not a syringyl unit (which has the 5-position blocked with a methoxyl group); thus levels are higher when relative syringyl levels are lower (S/G is lower). The correlations are visualized in Figure 6. Such correlations or associations can be powerful aids in enhancing our assignment capabilities in these complex cell wall samples. For example, the profile of two of the unassigned regions (ROI55 and ROI66) in the lignin region of the spectrum (Figure 1A) are highly positively correlated with H2/6 (r = 0.93, p < 0.0001 for both).

## Conclusions

The spectral dispersion inherent in 2D ^1^H–^13^C HSQC renders ROI segmentation methods useful for semi-quantitative studies of complex biological systems [21,22]. The profile of any single cross peak in the spectrum is linearly proportional to the concentration of the underlying species giving rise to the resonance. The term “semi-quantitative” is used here because the amplitude of different cross peaks in the 2D ^1^H–^13^C HSQC spectrum is not strictly comparable due to a range of factors relating to NMR methods themselves, and to the properties of the various polymers. For example the finite RF power available on the carbon channel in proton-carbon correlation experiments leads to non-uniform excitation of carbon resonances across the spectrum, although this is somewhat ameliorated by using adiabatic-pulse experiments [26]. If the experiment permits longer acquisition times, a range of quantitative 2D HSQC experiments [27,28] have been developed to mitigate this artifact.

We provide evidence here using a sizeable mutant study that FMLR reconstruction is useful both for rapid profiling of plant cell wall material and in improving the accuracy of conventional ROI segmentation methods for analysis of NMR spectra. The approach of generating a frequency domain spectrum from Fourier processing of a model time domain signal was used to reconstruct a model spectrum with close agreement to the processed data (Figure 2) using a small number of signals (degrees of freedom). An analysis of variance (ANOVA) in the SGH regions of the ROI feature matrix between pairs of mutant and wild-type sample groups yielded differences larger in magnitude using ROI segmentation coupled with FMLR reconstruction than with simple ROI integration alone. The difference between fixed-window integration techniques and spectral deconvolution is expected to be more pronounced in heterogeneous systems that display broad line widths such as in ball-milled preparations of plant cell wall material.

Even more significant is that assignment of ROIs to a mathematical model of the data rather than the data itself makes subsequent quantification less sensitive to changes in ROI definition. When modeled mathematically, the entire amplitude of a signal is assigned to an ROI as long as the peak center associated with the signal is encapsulated by the ROI. With direct integration of the spectrum itself, however, the ROI amplitude values are always modulated by changing the size or position of the ROI. This is an important consideration for general profiling using ROI segmentation because ROIs can be reused between studies with a minimal amount of adjustment (e.g., a constant ppm shift applied across all ROIs).

A strength of ROI segmentation methods is that prior information about spectral assignments can be used but is not required for profiling. In plant cell wall profiling, for example, the assignment of the lignin components is important not only in calculating SGH composition but also as a means of normalizing cross peaks from other regions of the spectrum. Even if a cluster of peaks is not assigned, the cluster may be associated with a region of interest and profiled across sample groups.

Conventional approaches create a feature set using spectral binning and then apply multivariate techniques to detect patterns among features across sample groups. The feature set of such an analysis is large and must eventually be related to a molecular species for targeted studies. This study provides an example of detecting patterns of enriched and depleted cell wall components using simple one-way ANOVA techniques directly on a meaningful feature set.

The analysis methodology has been implemented in a publicly-available, cross-platform (Windows/Mac/Linux), web-enabled software application (http://newton.nmrfam.wisc.edu) that enables researchers to view and publish detailed annotated spectra in addition to summary reports in standard csv formats. The csv format of the ROI feature matrix, for example, can be directly imported into dedicated software packages for metabolomic data processing and statistical analysis such as MetaboAnalyst 2.0 (http://www.metaboanalyst.ca) [29], as well as general statistical packages such as R (http://www.r-project.org/) and Matlab (http://www.mathworks.com/products/matlab/).

## Abbreviations

1D: 1-dimensional; 2D: 2-dimensional; 3D: 3-dimensional; 4CL: 4-coumarate: CoA ligase; 5-OH-G: 5-hydroxy-guaiacyl; ANOVA: Analysis of variance; C3H: *p*-coumarate 3-hydroxylase; C4H: Cinnamate 4-hydroxylase; CAD: Cinnamyl alcohol dehydrogenase; CCoAOMT: Caffeoyl-CoA *O*-methyltransferase; COMT: Caffeic acid *O*-methyltransferase; CCR: Cinnamoyl-CoA reductase; DMSO: Dimethyl-sulfoxide (-d_6_); DOE: (US) Department of energy; DP: Degree of polymerization; DSS: 4,4-dimethyl-4-silapentane-1-sulfonic acid (NMR standard); EPS: Encapsulated postscript; F5H: Ferulate 5-hydroxylase; FID: Free induction decay; FMLR: Fast maximum likelihood reconstruction; G: Guaiacyl; H: *p*-hydroxyphenyl; HCT: *p*-hydroxycinnamoyl-CoA:quinate/shikimate *p*-hydroxycinnamoyltransferase; HSQC: Heteronuclear single-quantum coherence (spectroscopy); NMR: Nuclear magnetic resonance (spectrometry); PAL: Phenylalanine ammonia lyase; Rms: Root-mean-square; ROI: Region of interest; ROIs: Regions of interest; S: Syringyl; SD: Standard deviation.

## Competing interests

The authors declare that they have no competing interests.

## Authors’ contributions

RAC developed the FMLR approach, its application to cell wall profiling, implemented the Newton software, and wrote the manuscript, RVA produced the *Arabidopsis* mutants, HK developed and ran the whole-CW NMR methodology, AA processed and prepared the NMR data in the required format, PM prepared and assisted with running the samples, JLM supervised RAC, VS aided in statistical analysis, WB conceived the *Arabidopsis* project and supervised RVA, and JR supervised PM, AA, and HK and aided in the analysis of the NMR spectra, manuscript writing, etc. All authors read and approved the final manuscript.
